# A comprehensive view on the apigenin impact on colorectal cancer: Focusing on cellular and molecular mechanisms

**DOI:** 10.1002/fsn3.3645

**Published:** 2023-08-28

**Authors:** Siamak Daneshvar, Mohammad Yasin Zamanian, Mehraveh Sadeghi Ivraghi, Maryam Golmohammadi, Mona Modanloo, Zahra Kamiab, Seyed Mohammad Ebrahim Pourhosseini, Mahsa Heidari, Gholamreza Bazmandegan

**Affiliations:** ^1^ Department of General Surgery School of Medicine Shahid Beheshti University of Medical Sciences Tehran Iran; ^2^ Department of Physiology School of Medicine Hamadan University of Medical Sciences Hamadan Iran; ^3^ Department of Pharmacology and Toxicology School of Pharmacy Hamadan University of Medical Sciences Hamadan Iran; ^4^ School of Medicine Qazvin University of Medical Sciences Qazvin Iran; ^5^ School of Medicine Shahid Beheshti University of Medical Sciences Tehran Iran; ^6^ Pharmaceutical Sciences Research Center Mazandaran University of Medical Sciences Sari Iran; ^7^ Clinical Research Development Unit Ali‐Ibn Abi‐Talib Hospital Rafsanjan University of Medical Sciences Rafsanjan Iran; ^8^ Department of Community Medicine School of Medicine Rafsanjan University of Medical Sciences Rafsanjan Iran; ^9^ Non‐Communicable Diseases Research Center Rafsanjan University of Medical Sciences Rafsanjan Iran; ^10^ Department of Internal Medicine School of Medicine Rafsanjan University of Medical Sciences Rafsanjan Iran; ^11^ Department of Biochemistry Institute of Biochemistry and Biophysics (IBB) University of Tehran Tehran Iran; ^12^ Physiology‐Pharmacology Research Center Research Institute of Basic Medical Sciences Rafsanjan University of Medical Sciences Rafsanjan Iran; ^13^ Department of Physiology and Pharmacology School of Medicine Rafsanjan University of Medical Sciences Rafsanjan Iran

**Keywords:** apigenin, apoptosis, colon cancer cells, PI3K/AKT/mTOR, STAT3

## Abstract

Colon cancer (CC) is one of the most common and deadly cancers worldwide. Oncologists are facing challenges such as development of drug resistance and lack of suitable drug options for CC treatment. Flavonoids are a group of natural compounds found in fruits, vegetables, and other plant‐based foods. According to research, they have a potential role in the prevention and treatment of cancer. Apigenin is a flavonoid that is present in many fruits and vegetables. It has been used as a natural antioxidant for a long time and has been considered due to its anticancer effects and low toxicity. The results of this review study show that apigenin has potential anticancer effects on CC cells through various mechanisms. In this comprehensive review, we present the cellular targets and signaling pathways of apigenin indicated to date in in vivo and in vitro CC models. Among the most important modulated pathways, Wnt/β‐catenin, PI3K/AKT/mTOR, MAPK/ERK, JNK, STAT3, Bcl‐xL and Mcl‐1, PKM2, and NF‐kB have been described. Furthermore, apigenin suppresses the cell cycle in G2/M phase in CC cells. In CC cells, apigenin‐induced apoptosis is increased by inhibiting the formation of autophagy. According to the results of this study, apigenin appears to have the potential to be a promising agent for CC therapy, but more research is required in the field of pharmacology and pharmacokinetics to establish the apigenin effects and its dosage for clinical studies.

## Graphical abstract

Apigenin is a natural flavonoid that has been studied for its potential anticancer effects, including in colon cancer. In summary, research of this study indicates that apigenin may have potential anticancer effects against colon cancer by: Inducing apoptosis and autophagy; Targeting specific signaling pathways such as STAT3 and NF‐B/Snail; Inhibiting EMT, migration, and invasion of colon cancer cells.

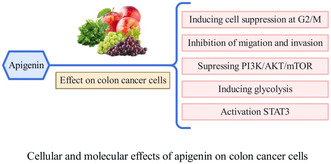

## 1 INTRODUCTION

Colon cancer (CC) ranks among the five most prevalent cancers in men and women (Argilés et al., 2020).

In the year 2020, around 1.9 million CC cases were registered, with the disease expected to increase by approximately 60% (~2.2 million) by the end of 2035 (Xi & Xu, 2021). The incidence of CC is intimately linked to the socioeconomic growth and lifestyle of the population in a given country (Condello & Meschini, 2021).

CC is a cancerous growth that originates from the colon's inner lining and can extend into deeper layers of the intestinal wall. Failure to treat CC can lead to serious health consequences (Jain et al., 2020). The risk factors for CC include age, family history, race, and lifestyle choices. Although some cases of CC are associated with genetic disorders, most are caused by aging and lifestyle habits (Cheng et al., 2022; Mishra et al., 2013). CC is responsible for about 10% of new cancer diagnoses worldwide and 9.4% of mortality associated with cancer, with an estimated 600,000 fatalities annually (Sung et al., 2021). Conventional treatments for CC, including surgery, chemotherapy, and radiation therapy, are frequently used by oncologists to cure this condition (Gündoğdu & Özyurt, 2023).

Chemotherapeutic and radiotherapeutic treatments are frequently used to combat tumors, but tumor cells can either possess or develop a natural tolerance to them (Xu et al., 2020).

This tolerance can result in high recurrence rates following surgery, which is associated with poor 5‐year survival rates.

Patients with stage I or II CC may experience partial or total colon resection surgery alone. In contrast, approximately two‐thirds of those with stage III disease typically receive neoadjuvant chemotherapy and colon resection surgery to mitigate the risk of CC recurrence (Miller et al., 2016).

Nevertheless, the potency of chemotherapeutic drugs is limited due to the development of resistance, which is a significant contributor to the poor clinical outcomes observed in treated patients. (Alfaleh et al., 2022). As a result, it is imperative to find new postoperative chemotherapy drugs that can increase the ability to kill cancer cells at lower doses (Printz, 2021).

Furthermore, traditional chemotherapy, as well as molecular targeted therapy, may cause toxicity in regular tissues. Therefore, it is crucial to develop new treatments that do not cause such problems for patients (Roshani et al., 2022).

There has been a growing interest in using traditional herbal medicines to create new drugs in recent years (Boroushaki et al., 2015; Dehnamaki et al., 2019; Zamanian et al., 2021). Research suggests that regular consumption of natural products can enhance patient well‐being (He et al., 2011; Patel et al., 2010; Shokrzadeh et al., 2018).

Flavonoids, which have antioxidant, antimutagenic, anti‐inflammatory, antiangiogenic, and anticancer properties, are fascinating (Mutha et al., 2021; Slika et al., 2022; Ullah et al., 2020; Zamanian et al., 2021).

They are considered one of the promising chemopreventive agents for cancer (de Sousa Silva et al., 2023; Ferreira et al., 2022).

**FIGURE 1:**
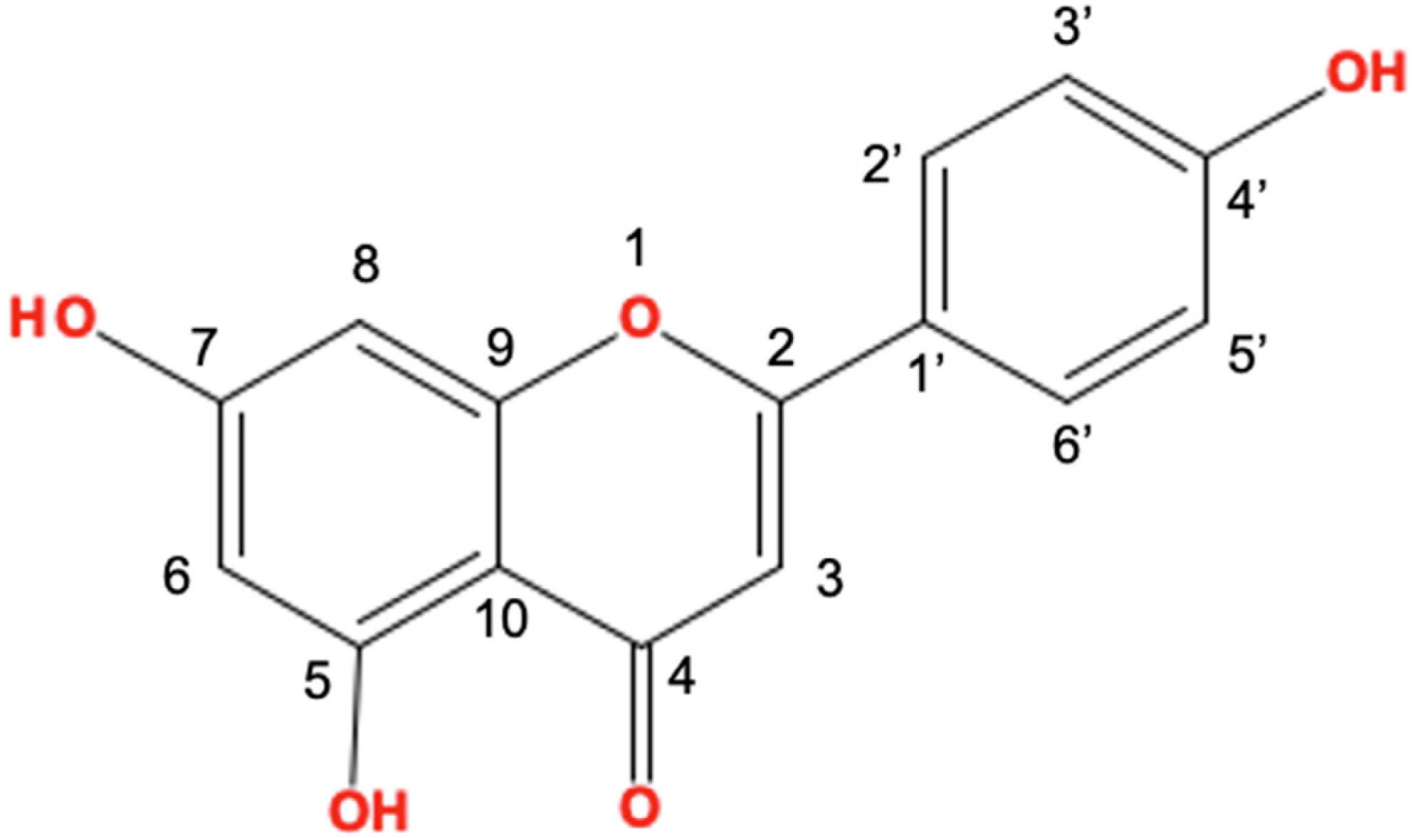
Chemical structure of apigenin.

Apigenin (4′, 5, 7‐trihydroxy flavone, Figure 1) is a biological compound belonging to the flavonoid subclass (Abid et al., 2022; Wang et al., 2019). It is a flavonoid compound that is commonly found in many fruits and vegetables, especially parsley, celery, celeriac, and chamomile tea (Mahbub et al., 2022; Mohammad Nabavi et al., 2015).

This compound is not poisonous and is plentiful in plant‐based foods. Apigenin has numerous probable biological activities, including antioxidant, anti‐inflammatory, and anticancer effects, and can concurrently exert multiple anticancer effects by modulating essential molecular targets (Jang et al., 2022; Li et al., 2022; Zafar et al., 2022).

Apigenin displays low cytotoxicity and significantly impacts cancer cells in reference to normal cells (Gupta et al., 2001). According to various studies, apigenin downsizes cell proliferation and causes apoptosis in CC cells (Lee et al., 2014; Turktekin et al., 2011).

Apigenin shows an oncolytic effect through apoptosis induction by modulating the quantity of caspases, BAX, Bcl2, p53, etc (Mabrouk Zayed et al., 2022). Apigenin also regulates cell cycle advancement by stopping the cycle arrest at G2/M or G0/G1 checkpoint (Pandey et al., 2023). In addition, it promotes autophagy, hinders migration and invasion, and deters angiogenesis (Chen et al., 2019). Regarding the molecular anticancer mechanism of apigenin, it controls the PI3K/AKT/mTOR signaling pathway, NF‐κB/MAPK/ERK pathway, and Wnt/JNK pathway (Ahmed et al., 2021).

Thus, the aim of this review is to depict the role of apigenin in the treatment of CC in a cellular and molecular manner.

## 2 OVERVIEW OF APIGENIN

### 2.1 Structure and summary of mechanisms

Flavones are in a flavonoid class with a 2‐phenylchromen‐4‐one (2‐phenyl‐1‐benzopyran‐4‐one) backbone (Dajas et al., 2013; Ueda et al., 2004).

Apigenin, a natural flavone, is abundant in diverse plant‐based foods and beverages. It is common in parsley, grapes, red wine, chamomile tea, and apples. Typically, apigenin is conjugated to a glycoside (Jang et al., 2022; Jangdey et al., 2018).

The major source plants of apigenin include Tanacetum, Artemisia, Matricaria genera, and Achillea pertaining to the Artemisia family of plants (Ornano et al., 2016; Sharifi‐Rad et al., 2018; Venditti et al., 2015, 2018).

Certain plants contain apigenin in two forms: as aglycone or as different types of apigenin glycosides, such as apigenin‐7‐O‐glucoside, apigenin‐6‐C‐glucoside (also known as isovitexin), apigenin‐8‐C‐glucoside (also known as vitexin), apigenin‐7‐O‐neohesperidoside (also known as rhoifolin), and apigenin‐6‐C‐glucoside‐8‐C‐arabinoside (Nabavi et al., 2018). A list of various dietary sources of apigenin can be found in Table 1 (Thomas et al., 2023).

**TABLE 1:** Various dietary sources of apigenin.

Sources	Glycoside	Quantity (mg/100 g or mg/100 mL)
Olive oil, extra virgin	Apigenin aglycone	1.17
Italian oregano, fresh	Apigenin aglycone	3.50
Globe artichoke, heads, raw	Apigenin 7‐O‐glucuronide	7.40
Orange, pure juice	Apigenin 6, 8‐di‐C‐glucoside	5.53
Kumquats	Apigenin 7‐O‐neohesperidoside	21.87

The synthesis of apigenin takes place on the exterior of the reticular apparatus through a four‐step process involving the synthesis of the intermediate, basic skeleton, precursor, and apigenin structure (Seo et al., 2011). Contemporary data suggest that the chemical structure of apigenin is critical to its bioactivity. By removing molecular subunits linked to specific biological activities, researchers can determine the structure–activity relationship of apigenin. For instance, the blocking of α‐glucosidase and α‐amylase is attributed to the presence of double bonds in the two aromatic rings and hydroxyl groups on C‐7 and C‐4′ (Li et al., 2018). On the other hand, the immune system regulation of apigenin requires a hydroxyl group at C‐4′ in ring B (Kilani‐Jaziri et al., 2016). Finally, OH radicals at positions 5, 7, and 4′ are critical in activating the Liver X receptor (Fouache et al., 2019).

Apigenin has been used as a conventional drug for many years on account of its antioxidant nature (Zafar et al., 2022) and inflammatory properties (Li et al., 2022), and anticancer outcomes (Rahmani et al., 2022; Zhou et al., 2022).

Previous research indicates that it contains broad anticancer properties; for instance, pancreatic, breast, colorectal, and prostate (Feng et al., 2023; Hnit et al., 2022; Hong et al., 2022; Qu et al., 2022). Apigenin works by inhibiting tumor cell proliferation through the induction of cell apoptosis and autophagy and modulating the cell cycle (Liang et al., 2023).

Several studies have investigated the effects of apigenin on metastasis and have found that it can inhibit the process in various types of cancer cells. Apigenin was found to suppress bone metastasis of human breast cancer cells by inducing apoptosis, autophagy, and modulation of the MEK/ERK signaling pathway (Wu et al., 2020). Apigenin inhibited migration and metastasis of human hepatocellular carcinoma (HCC) cells by suppressing the NF‐κB/Snail pathway and reversing increases in epithelial–mesenchymal transition (EMT) marker levels (Qin et al., 2016).

Additionally, it has been shown to control the immune response and the activity of NF‐κB, particularly in the lungs (Cardenas et al., 2016). In the process of tumor inhibition, apigenin modulates a couple of protein kinases and signaling pathways, including MAPK/ERK, PI3K/Akt, JAK/STAT, NF‐κB, and Wnt/β‐catenin (Yan et al., 2017).

Apigenin has been shown to have a significant part in causing apoptosis. This is accomplished by triggering the MAPK signaling pathway and reducing sulfiredoxin expression (Wang et al., 2022). Studies have demonstrated that apigenin increases the cleavage of poly‐(ADP‐ribose) polymerase (PARP) and rapidly enhances caspase‐3 activity, resulting in DNA fragmentation and apoptosis (Ghanbari‐Movahed et al., 2023; Gupta et al., 2002). These effects are attributed to a shift in the Bax/Bcl‐2 ratio in favor of programmed cell death.

According to research, apigenin can hinder the development of lung tumor cells and transcriptional activation of vascular endothelial growth factor (VEGF) in a concentration‐dependent style. The means by which apigenin suppresses VEGF transcription is indicated to be associated with the decrease in hypoxia‐inducible factor 1‐alpha (HIF‐1α; Jin & Ren, 2007).

It has also been indicated that apigenin can enhance autophagy and activate cell death in primary effusion lymphoma while significantly lowering the level of reactive oxygen species (ROS). Additionally, apigenin can activate p53, which improves catalase and inhibits STAT3, as demonstrated by p53 silencing experiments (Granato et al., 2017).

The use of apigenin caused the arrest of lung tumor cells at the G2/M phase of the cell cycle. Treating patients with apigenin was found to increase p53 and p21CIP1/WAF1 (Lee et al., 2014). Apigenin also enhanced cellular cycling and cell suicide by hindering the PI3K/Akt/mTOR pathway (Yang et al., 2018). Scientists have discovered that apigenin has the prospect of preventing cancer by controlling the ERK1/2 MAPK and PI3K/Akt signaling pathways, thus stopping the growth and spread of tumors (Lim et al., 2016). In addition, by inhibiting the Wnt/β‐catenin pathway, apigenin significantly reduced the growth, and spread of malignant cells, their ability to divide, invade surrounding tissues, migrate to new areas, and form organoids (Xu et al., 2016).

### 2.2 Bioavailability of apigenin

Including natural products derived from plants is recommended as part of prophylactic and treatment strategies for disease management (Chupradit et al., 2022; Zamanian et al., 2021).

Many of the therapeutic effects of therapeutic plants are attributed to different phytochemicals including flavonoids or active compounds (Mutha et al., 2021; Ullah et al., 2020).

Bioavailability is a crucial factor in drug delivery, referring to the amount and rate of the first dose of a prescription that reaches the target site or access the body (Herkenne et al., 2008; Thilakarathna & Rupasinghe, 2013). Hydrophobic compounds generally exhibit poor bioavailability because of limited capability of absorption, resulting in low levels of the drug reaching the target tumor and advanced toxicity to normal tissues (Darakhshan et al., 2015). Bioavailability can also be described as the amount of a compound absorbed, digested, metabolized, and excreted after food is ingested through the mouth (Rein et al., 2013). Typically, when medicine is taken through the mouth, polyphenols are mainly taken up and processed in the small bowel (Kahle et al., 2007), with only a small fraction being absorbed in the stomach (Crespy et al., 2002).

The hydrophilic properties of apigenin range from 0.001 to 1.63 mg per milliliter in nonpolar solvents (Mohammad Nabavi et al., 2015), in contrast, the solubility in a phosphate buffer is 2.16 μg/mL (Zhang et al., 2012). Apigenin is not easily absorbed orally because of its low water solubility, which is only 2.16 g/mL (Salehi et al., 2019), significantly hindering its clinical development. Apigenin is slowly absorbed and eliminated from the body, as evidenced by its half‐life of 91.8 h in the blood, a distribution volume of 259 mL, and a plasmatic clearance of 1.95 mL/h (Gradolatto et al., 2005).

However, a novel mesoporous silica nanoparticle drug carrier has been shown to improve the ability of apigenin to dissolve, be absorbed orally, and become bioavailable in the body (Huang et al., 2019).

### 2.3 Cytotoxicity of apigenin

Apigenin, a plant‐derived compound, has demonstrated selective anticancer effects and effective cell cytotoxic activity while exhibiting negligible toxicity to ordinary cells. It has also been found to quash cancer stem cells (CSCs; Ketkaew et al., 2017). The purpose of autophagy in the cytotoxic effect caused by apigenin varies depending on the type of cancer cell. Many studies suggest that autophagy induced by apigenin is responsible for developing resistance to cell apoptosis in cancer cells. This has been shown by the fact that combining apigenin with autophagy inhibitors increases cell apoptosis, indicating that autophagy reduces the cytotoxic effects of apigenin on cancer cells. However, in human papillary thyroid carcinoma BCPAP cells, autophagy leads to autophagy‐mediated cell demise (Kim et al., 2015). A study employing the use of 3‐MA, an autophagy suppressor, significantly augmented the level of apoptosis induced by apigenin, indicating that apigenin's autophagy‐mediated tumor protective properties are still present (Cao et al., 2013). Therefore, the function of autophagy in apigenin‐induced cytotoxicity depends on the type of cancer cell, as autophagy helps cancer cells to protect themselves from the cytotoxic effects of apigenin in some cases (Yan et al., 2017).

### 2.4 Apigenin as an anticancer agent

Anticancer drugs often have numerous harmful side effects that can cause significant harm to the organs of the patients and exacerbate their symptoms. (Schirrmacher, 2019). As such, it is essential to comprehend and be aware of these toxicities and adverse effects, especially with the rising prices of drugs and the reduction in the quantity of medications that are genuinely effective and approved by the FDA. Synthetic small molecule compounds have been found to have acquired drug resistance and negative effects, prompting the search for flavones, which are believed to have significant physiological usefulness as they have low toxicity and are not mutagenic in humans (Slika et al., 2022). Apigenin, a flavone, has gained much traction in developing anticancer agents. It has been demonstrated to have potential as a dietary supplement or a supplementary chemotherapeutic agent for treating cancer (Rahmani et al., 2022). Studies have shown that apigenin displays notable cytotoxicity toward different kinds of cancer cells and suppresses CSCs in an assortment of cancers, making it an attractive candidate for further investigation in cancer treatment. Apigenin has also been found to induce cell cycle arrest, trigger cell apoptosis and autophagy, stimulate an immune reaction, and suppress metastasis and aggression in multiple human cancers in vitro and in vivo via various biological mechanisms (Pandey et al., 2023; Yan et al., 2017; Zhong et al., 2021; Zhou et al., 2022).

### 2.5 Possible side effects of apigenin

Apigenin is generally considered safe for intentional consumption in higher doses, as the toxicity hazard is low. Apigenin quantity in a person's diet is not likely to get to a level that would cause injury (Shao et al., 2013).

However, intentional consumption of high doses of dietary supplements may result in a slightly increased risk of experiencing adverse effects. Some of the probable adverse reactions include stomachache, muscle relaxation, as well as sleepiness (Abid et al., 2022).

Consuming chamomile extract, which is high in apigenin, may cause stomach discomfort, and it is recommended to discontinue its use if this occurs (Abid et al., 2022). Some people may experience skin irritation when using topical products containing apigenin. It is advisable to consult a healthcare provider before taking apigenin supplements if a person is taking prescription medications due to the risk of drug interactions. The risk is particularly high for those taking cyclosporine, warfarin, or certain types of chemotherapy drugs (Pham et al., 2012).

## 3 EFFECTS OF APIGENIN ON COLON CANCER

CC is a predominant gastrointestinal cancer with high death rates and increased morbidity in recent years (Zhang et al., 2023). Ineffective treatment interventions and late disease recognition at progressive stages make this cancer a difficult area to manage (Gupta et al., 2019). The use of anticancer drugs for CC treatment is associated with significant side effects that negatively affect patient quality of life (Mao et al., 2011). In this regard, natural plant‐derived molecules have gained significant attention due to their comparatively lower toxicity. Researchers worldwide are focused on identifying and screening natural products against cancer cells to develop efficient drugs that can target colon and other cancers (Yang et al., 2012).

Autophagy and apoptosis are cellular functions that play important roles in maintaining tissue equilibrium and eliminating unwanted or harmful cells, including cancer cells (Amaravadi et al., 2019; Nazio et al., 2019).

The PI3K/AKT/mTOR signal transduction pathway is often anomalously triggered in cancer cells and is involved in their proliferation (Barrett et al., 2012).

In a study on cisplatin‐resistant CC cells, apigenin was found to have significant anticancer effects at doses of 15, 30, and 60 μM. The anticancer action of apigenin was credited to its capacity to cause both autophagy and programmed cell death. Additionally, apigenin was shown to inhibit the PI3K/AKT/mTOR signal transduction pathway in cisplatin‐resistant CC cells (HT‐29 cells). In vivo studies in mice also revealed that apigenin (35 mg/kg dose) hindered the development of xenografted tumors (Chen et al., 2019).

Caspase‐3, an executioner caspase, serves an indispensable function in apoptosis and is a primary target for cancer therapy (Yadav et al., 2021). Caspase‐8 is an apical caspase that initiates programmed cell death in response to the ligation of the death receptor. This essential involvement in apoptosis has sparked tremendous therapeutic interest in controlling caspase‐8 expression and proteolytic activity (Stupack, 2013).

Turktekin et al. observed that a 100 μM concentration of apigenin could force cellular death in CC cells and target multiple molecular pathways involved in the disease's progression. The study also showed that apigenin upregulated caspase‐3 and caspase‐8 while downregulating mTOR and cyclin D1. Interestingly, the researchers found that apigenin induced a p53‐independent caspase cascade, indicating that it can target CC cells that have mutated p53. Finally, the decline in mTOR expression levels suggested that apigenin drives CC cells toward apoptosis rather than autophagy (Turktekin et al., 2011).

In a separate investigation, Lee et al. found that apigenin (25 and 50 μM) impeded the development of HCT116 cells by inducing cell cycle suppression at the G2/M phase. Furthermore, apigenin therapy leads to increased apoptosis as well as autophagy. The use of the autophagy inhibitor, 3‐methyladenine (3‐MA), improved the apoptosis triggered by apigenin by activating pro‐caspases‐8, ‐9, and ‐3, as well as cleaving poly (ADP‐ribose) polymerase (PARP). These findings suggest that suppressing autophagy may be an effective approach to enhance the effectiveness of chemotherapy for CC treatment using anticancer agents (Lee et al., 2014).

Epithelial–mesenchymal transition (EMT) is a crucial process that plays a role in secondary tumor formation and plays a significant role in CC advancement (Bellovin et al., 2005).

Targeting EMT can be helpful for the prediction and treatment of CC (Bates & Mercurio, 2005; Lefort & Blay, 2011). Some regulatory proteins, for instance, the Snail/Slug family of zinc‐finger proteins and Twist Family BHLH Transcription Factor 1 (Twist1), regulate EMT (Yu et al., 2010).

Research indicates that the NF‐κB pathway is involved in controlling EMT in malignant cells by triggering Snail transcription (Chakrabarti et al., 2004; Julien et al., 2007; Liu et al., 1999). The NF‐κB pathway controls Snail expression through transcriptional and posttranslational mechanisms and boosts Snail transcription (Barbera et al., 2004).

According to Tong et al., apigenin showed curtailing impacts on the metastasis and aggression of colon carcinoma cells. They found that apigenin at loads of 10 and 20 μM suppressed EMT of HCT‐116 and LOVO human CC cells via the NF‐κB/Snail signaling pathway. Furthermore, the effectiveness of apigenin (200 and 300 mg/kg) in treating CC was evaluated by establishing xenografts on Balb/c nude mice. It is worth noting that the use of the PDX (patient‐derived xenografts) model may better reflect the properties of clinical tumor specimens (Tong et al., 2019).

Apoptosis is a regulated cell death process that removes impaired or dangerous cells (Carneiro & El‐Deiry, 2020). The Bcl‐2 proteins regulate apoptosis, including pro‐apoptotic proteins (Bax and Bak) and antiapoptotic proteins (Bcl‐xL and Mcl‐1; García‐Navas et al., 2021; Zamanian et al., 2017). The balance between these proteins can be altered by downregulating antiapoptotic proteins or upregulating proapoptotic proteins, leading to the discharge of cytochrome c and triggering the caspase cascade (Wang & Youle, 2009).

Bcl‐2 is a crucial inhibitor of apoptosis, and its overexpression is associated with malignant transformation and chemotherapy resistance (Burlacu, 2003; Warren et al., 2019). Thus, targeting the Bcl‐2 proteins, especially the antiapoptotic proteins, may represent a promising approach for cancer treatment.

Myeloid cell leukemia 1 (Mcl‐1), a prosurvival Bcl‐2 protein, is commonly overexpressed in cancers that affect humans (Tong et al., 2017).

This protein hinders cell apoptosis by attaching itself to proapoptotic Bcl‐2 proteins, thereby repressing MOMP and caspase activation (Perciavalle & Opferman, 2013). Mcl‐1 has been identified as a critical factor in tumor cell survival and resistance to therapy (Perciavalle & Opferman, 2013).

Recent research shows that apigenin decreases the expression of Mcl‐1 in CC cells (Shao et al., 2013).

Multiple analyses show that STAT proteins, for instance, STAT3, display persistent activity in various human cancer cells and promote the advancement of cancer (Lee et al., 2019; Tolomeo & Cascio, 2021).

In colorectal cancer, sustained activation of STAT3 is linked to increased cellular multiplication and invasion in vitro and tumor growth in vivo, while blocking STAT3 activity can induce apoptosis and reduce cancer cell invasion (Lin et al., 2011; Lu et al., 2016; Wang et al., 2013).

Previous research has suggested that apigenin can suppress tumor growth in various cancers by targeting the STAT3 signaling pathway (Cao et al., 2016; Seo et al., 2015).

**FIGURE 2:**
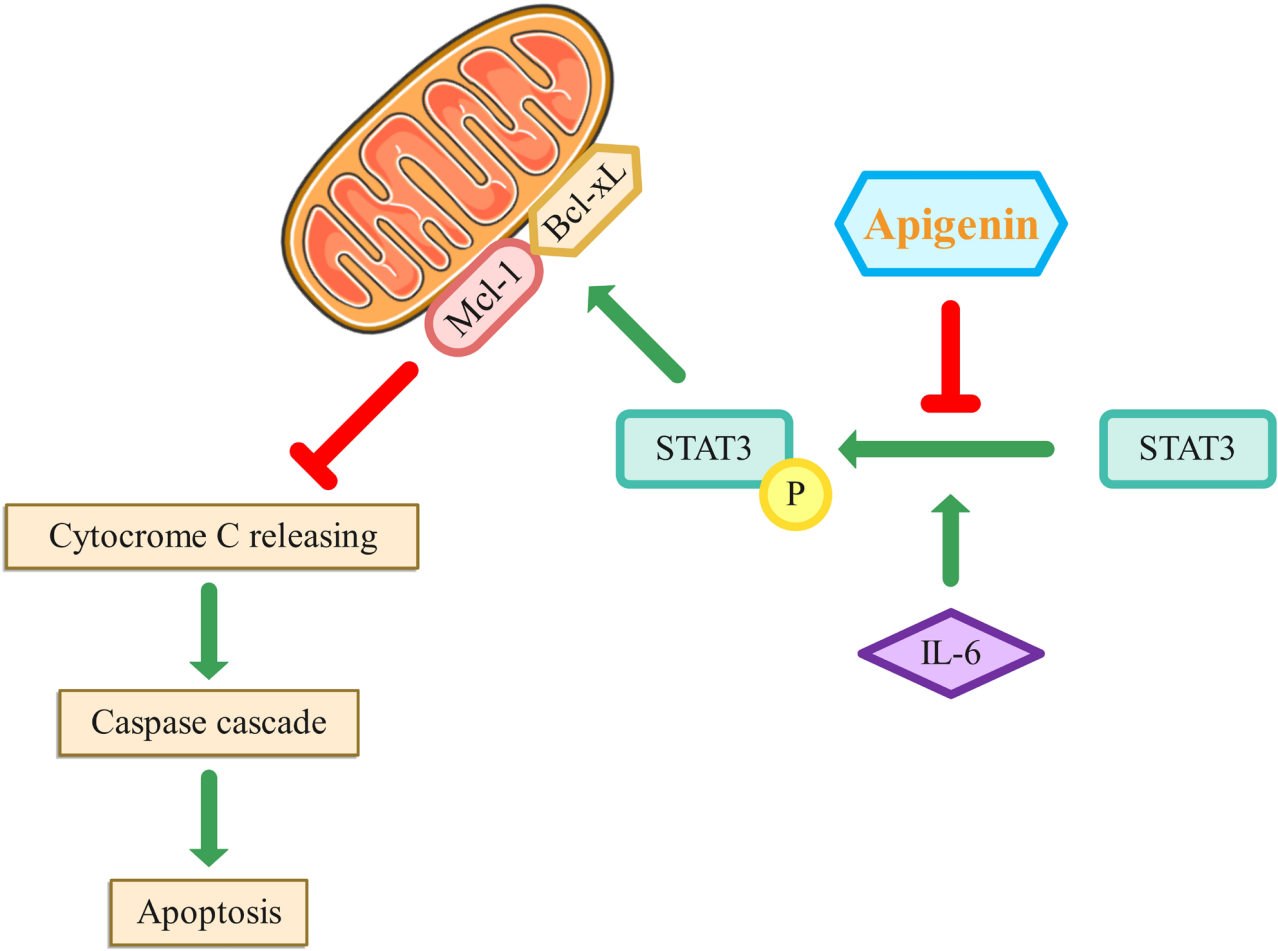
The mechanism of action of apigenin on the STAT3 signaling pathway and the Bcl‐xL/Mcl‐1 axis in CC cells. In CC, the overexpression of Bcl‐xL and Mcl‐1 contributes to the survival and proliferation of cancer cells by inhibiting apoptosis. The upregulation of these proteins helps cancer cells evade cell death signals and promotes tumor growth and resistance to therapy. It was found that treatment with IL‐6 increased the expression of p‐STAT3, Bcl‐xL, and Mcl‐1 in CC cells. Additionally, apigenin treatment resulted in a dose‐dependent decrease in the levels of p‐STAT3 in CC cells. Cytochrome c is a critical mediator of apoptosis. Its release from the mitochondria triggers the activation of caspases and initiates the apoptotic process. The balance between pro‐apoptotic and antiapoptotic Bcl‐2 family members determines whether cytochrome c is released and apoptosis is induced.

Maeda et al. discovered that apigenin (50 μM) causes death in CC cells (DLD‐1 and HCT116 cell lines) by targeting two antiapoptotic proteins, Bcl‐xL as well as Mcl‐1, through STAT3 pathway of cellular signaling. The study showed that IL‐6 stimulation increased p‐STAT3 expression, Bcl‐xL, and Mcl‐1, but administration of apigenin decreased their expression. Furthermore, concurrent inhibition of Bcl‐xL and Mcl‐1 led to increased deaths of CC cells, indicating that these two proteins are essential medicinal targets of CC. These findings suggest that apigenin may be designed as an effective therapeutic agent for CC by concurrently targeting and repressing the expression of Bcl‐xL and Mcl‐1. Figure 2 illustrates the mechanism of action of apigenin on the STAT3 signaling pathway and the Bcl‐xL/Mcl‐1 axis in CC cells (Maeda et al., 2018). Maeda et al., (2018) found that apigenin inhibited the phosphorylation of STAT3 in a dose‐dependent manner, as the levels of p‐STAT3 significantly decreased with increasing concentrations of apigenin. This suggests that apigenin has an inhibitory effect on STAT3 phosphorylation in CC cells.

NF‐κB is a well‐researched proinflammatory transcription factor, but research indicated its significant inclusion in the development of CC and other types of cancer by promoting neovascularization, metastasis, proliferation, and inhibiting apoptosis (Slattery et al., 2018).

In a pharmacokinetic study, rats administered with 60 mg/kg of apigenin showed a Cmax of 1.33 ± 0.24 μg/mL and an AUC0–t° of 11.76 ± 1.52 μg h/mL (Ding et al., 2014). However, to overcome this limitation, researchers developed apigenin‐loaded lipid–polymer HyNP (LPHyNPs) and evaluated its cytotoxic potential in an in vitro model of CC.

In an analysis conducted by Alfaleh et al. NF‐κB expression level was analyzed following treatment with different formulations. The outcome showed that treatment with LPHyNPs significantly reduced the expression level of NF‐κB compared to blank LPHyNPs in HCT 116 cells. The study also investigated the anticancer properties of LPHyNPs through regulation of the expression of mTOR, JNK, and MDR‐1. Results indicated a noteworthy decrease in mTOR and JNK expression levels with LPHyNPs treatment in comparison with blank LPHyNPs (Alfaleh et al., 2022).

Navitoclax, also known as ABT‐263, is an orally accessible small chemical that inhibits Bcl‐2 selectively and potently (Rudin et al., 2012).

Shao et al. discovered that apigenin (20 μM) enhances the anticancer effects of Navitoclax or ABT‐263, an orally bioavailable small molecule that selectively and potently inhibits Bcl‐2 in various colon cancer cells, including DLD1, HCT116, HCT‐8, HT29, and SW48, by controlling the expression of Mcl‐1 and other pro‐survival effectors. The study then evaluated the antitumor efficacy of ABT‐263 (100 mg/kg) and apigenin (25 mg/kg) in mice. Immunoblotting of tumor tissues revealed that treatment with apigenin or a combination of apigenin and ABT‐263 resulted in decreased expression of Mcl‐1 and reduced phosphorylation of AKT and ERK, suggesting that apigenin can also target these pro‐survival modulators in vivo (Shao et al., 2013).

The Warburg effect, aerobic glycolysis, is noted in neoplastic cells, where glucose is converted into lactate rather than undergoing complete oxidation via the Krebs cycle, even in sufficient oxygen (Ryan et al., 2019).

This shift toward glycolysis is necessary for tumor cell proliferation and growth, as it is the major energy‐generating pathway for these cells (Vander Heiden et al., 2009).

The enzyme pyruvate kinase (PK) facilitates the ultimate rate‐controlling phase of glycolysis (Christofk et al., 2008).

Pyruvate kinase isozymes M1 (PKM1) and PKM2 are ciphered by one PK gene, while another gene encodes PKL and PKR. PKM2 is uniformly depicted in most malignant cells and occupies a key position in cancer progression as a controller of aerobic glycolysis (Chen et al., 2011; Li et al., 2014). Therefore, identifying medications that can hamper PKM2 activity and expression may be an innovative approach to antineoplastic treatment.

Glucose transporter 1 (GLUT1) is a protein that facilitates the transport of glucose across cell membranes and is involved in glucose uptake and metabolism (Pascual et al., 2004). It is expressed in many cell types, including cancer cells, and plays a key role in the Warburg effect and cancer metabolism (Young et al., 2011; Zhang et al., 2015). Apigenin has been found to inhibit GLUT1 activity and glucose uptake in human pancreatic cancer cells (Melstrom et al., 2008). Therefore, it may have the same effect on CC cells.

Polypyrimidine tract binding protein (PTBP1) is an important controller of the splicing of PK genes that selectively promotes the expression of PKM2 (Calabretta et al., 2016).

A study by Minami et al., (2017) showed that c‐Myc, an oncogenic transcription factor, can positively regulate the expression of PTBP1 in malignant cells.

β‐catenin, another protein, is known to be upstream of c‐Myc and can upregulate transcription of PTBP, thereby guaranteeing a high PKM2/PKM1 ratio (David et al., 2010; Noubissi et al., 2006).

**FIGURE 3:**
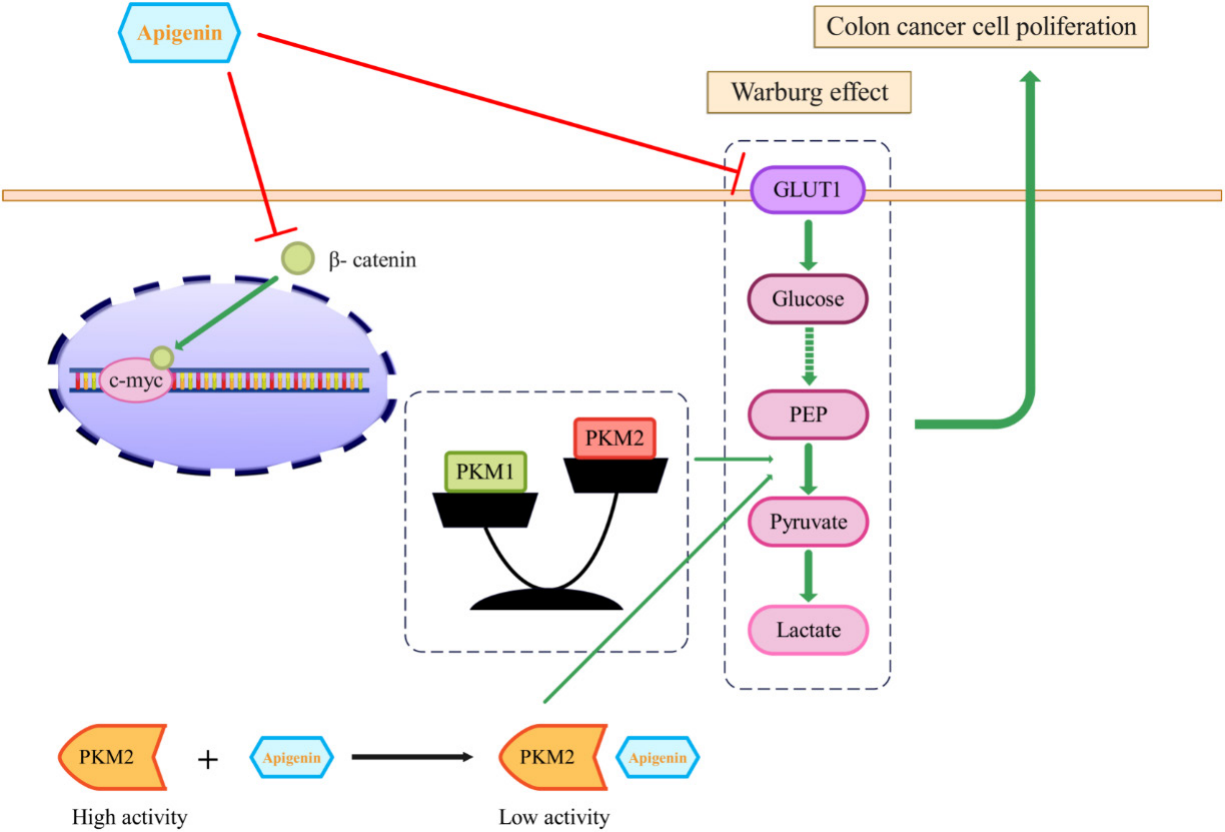
Effects of apigenin on CC cell proliferation via targeted blocking of PKM2‐dependent glycolysis. Apigenin could inhibit the activity and expression of PKM2, which is the last rate‐limiting enzyme in glycolysis. In addition to its effects on PKM2, apigenin also influenced the expression of PKM1. The regulation of PKM2 and PKM1 expression by apigenin was mediated through the β‐catenin/c‐Myc signaling pathway. Apigenin significantly reduced the expression of β‐catenin and c‐Myc, which are downstream target genes of β‐catenin. Overall, these findings suggest that apigenin may play a crucial role in inhibiting CC cell proliferation by targeting the PKM2‐mediated glycolysis pathway and inducing a switch from glycolysis to oxidative phosphorylation.

According to Shan et al., apigenin (15 and 30 μM) inhibited the activity of PKM2 in CC cells (HCT116, HT29, and DLD1). The study revealed that AP can directly bind to PKM2, leading to a significant inhibition of its activity and expression. This inhibition of PKM2 activity by apigenin ultimately blocks the glycolysis pathway in colon cancer cells. It was also observed that apigenin acts as an allosteric inhibitor of PKM2, as its inhibitory effect on PKM2 was not reversed even in the presence of D‐fructose‐1,6‐diphosphate (FBP), which is a known activator of PKM2. Furthermore, apigenin was found to regulate the PKM2/PKM1 ratio in CC cells by blocking the β‐catenin/c‐Myc/PTBP1 signal pathway. This regulation ensures a low PKM2/PKM1 ratio, which is associated with the suppression of glycolysis and the inhibition of cancer cell proliferation. Overall, the specific targeting and inhibition of PKM2 activity by apigenin in colon cancer cells highlight the potential of apigenin as a novel therapeutic strategy for CC treatment (Shan et al., 2017). Figure 3 illustrates the mechanism of action of apigenin on the PKM2 and PKM1 in CC cells.

Adenomatous polyposis coli (APC) is a well‐known antioncogene, and loss of both alleles is required for the initiation of tumorigenesis (Kariv et al., 2020).

Chung et al. demonstrated that apigenin (80 μM) arrested cell cycle progression at the G2/M phase and inhibited cell growth in HT29‐APC and HT29‐GAL cells. In HT29‐APC cells induced with zinc, which expressed wild‐type APC gene, apigenin failed to prompt G2/M the halting of cellular replication, indicating that it may be more efficient in causing G2/M arrest in cells with APC mutations. Interestingly, apigenin significantly increased the expression of APC in HT29‐APC cells induced with zinc, which was linked with boosted noninflammatory CC cell death. These findings imply that apigenin might boost cellular death in CC cells with wild‐type APC and stimulate cell cycle arrest in CC cells with mutant APC (Chung et al., 2007).

Protein kinase CK2 is a serine–threonine kinase known to be disrupted in different human cancers (Trembley et al., 2022).

Colon cancer progression is often accompanied by inflammation, where many inflammatory cytokines are upregulated, including tumor necrosis factor‐alpha (TNF‐α). This cell signaling protein leads to necrosis and inflammation (Lai et al., 2020; Zhao & Zhang, 2018).

Farah et al. performed research demonstrating the effectiveness of two blockers of CK2, 5,6‐dichloro‐ribifuranosylbenzimidazole (DRB), and apigenin (7.5 μM), combined with TNF‐α, leading to a collaborative decrease in cell survival HCT‐116 and HT‐29 cells. Additionally, treatment with either DRB or apigenin decreased MnSOD expression when stimulated with TNF‐α in HCT‐116 CC cells. These findings suggest that these inhibitors may be a viable treatment option for CC (Farah et al., 2003). These findings suggest that apigenin could be a promising therapeutic agent for CC treatment. However, further study is needed to fully understand its mechanisms and efficacy in clinical studies. The results of the studies are summarized in Table 2.

**TABLE 2:** Some studies consist of the purpose of the present review.

Authors	Dosage	Type of study/model	Mechanisms
Chen et al.	15, 30, and 60 μM 35 mg/kg	HT29 cells mice	Induces autophagy and programmed cell death inhibits the growth of xenografted tumors
Turktekin et al.	100 μM	HT29 cells	Increases caspase‐3 and caspase‐8 activity
Lee et al.	25 and 50 M	HCT116 cells	Inhibits the G2/M stage of the cell cycle
Maeda et al.	50 μM	DLD‐1 and HCT116 cell lines	Reduces the expression of p‐STAT3, Bcl‐xL, and Mcl‐1
Chung et al.	80 μM	HT29‐APC and HT29‐GAL cells	Arrests cell cycle progression at the G2/M phase and inhibits cell growth
Shao et al.	25 mg/kg	Severe combined immunodeficient (SCID) Mice	Reduces the expression of Mcl‐1 and the phosphorylation of AKT and ERK in tumor tissues
Farah et al.	7.5 μM	HCT‐116	Decreases MnSOD expression

## 4 CONCLUSION

**FIGURE 4:**
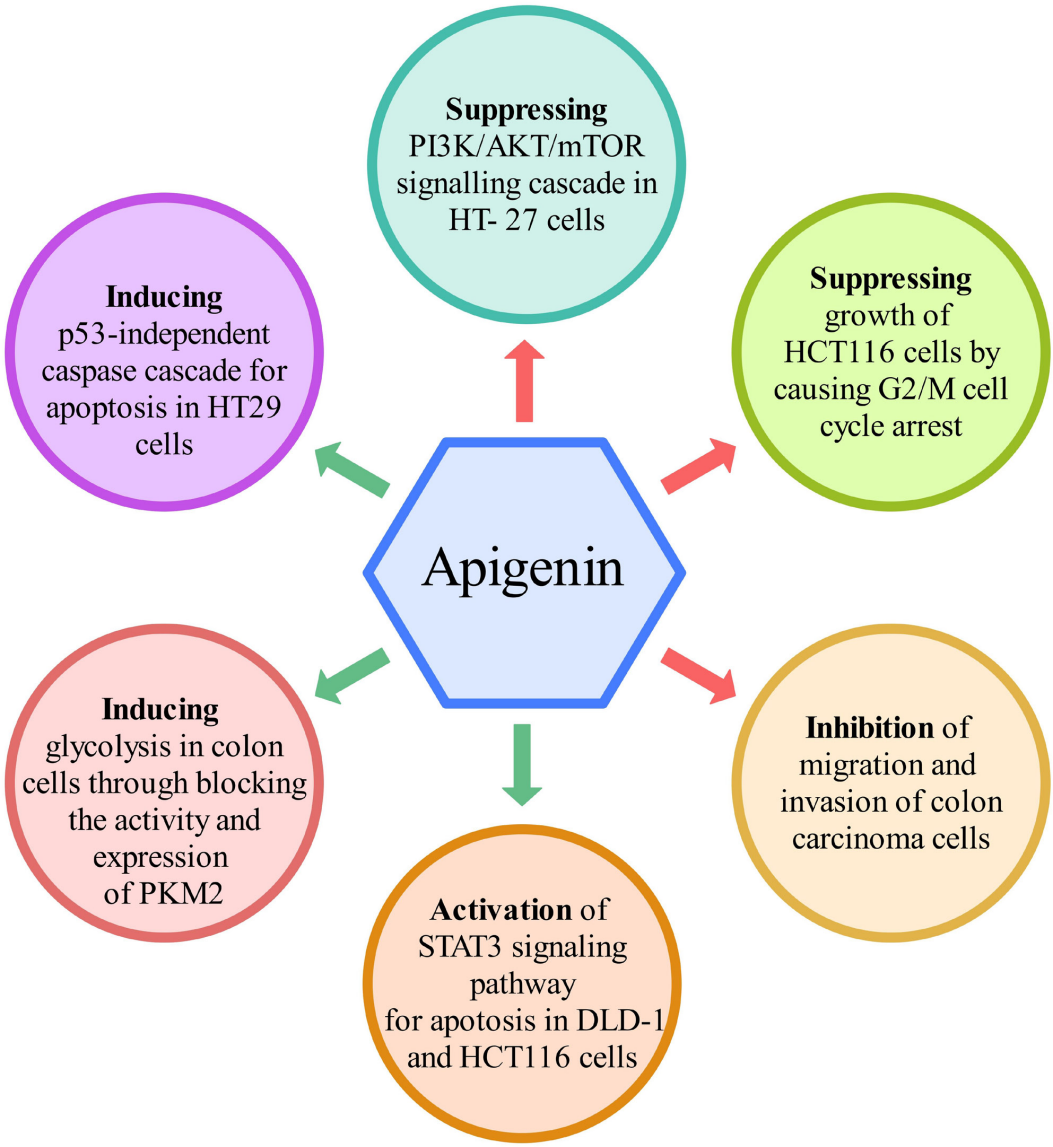
The effects of apigenin on CC cells.

Apigenin as a flavonoid has shown promise in treating various types of cancer, including colon, pancreatic, breast, colorectal, and prostate cancer cells. Studies have demonstrated that combination therapy with apigenin in different types of cancers not only enhances the efficacy of chemotherapy but also reduces the side effects by targeting multiple cell signaling pathways. Apigenin has been reported to suppress CC in vitro and in vivo by multiple biological effects, such as triggering cell apoptosis and autophagy, inducing cell cycle arrest, and suppressing cell migration and invasion. We summarized the effects of apigenin on CC cells in Figure 4. Taken together, these results indicate that apigenin could inhibit the growth of CC cells in vitro and in vivo and may be used for the improvement of therapy for colon cancer.

## AUTHOR CONTRIBUTIONS

M.Y.Z, M.M, and Gh.B: conception, design, writing, and revising the manuscript. S.D and M.H.: revising and editing the manuscript and graphic drawing. M.G, M.S.I, and Z.K: data gathering and editing the manuscript. S.M.E.P, S.D, and M.H. contributed to data collection, drafting of the manuscript, and table creation. All authors read and approved the final manuscript.

## FUNDING INFORMATION

None.

## CONFLICT OF INTEREST STATEMENT

The authors declare that they have no conflict of interest.

## ETHICS STATEMENT

This article does not contain any studies with human participants or animals performed by any of the authors.

## Data Availability

The data relevant to the review article are within the manuscript.
